# Large Intervening Non-Coding RNA *HOTAIR* Is an Indicator of Poor Prognosis and a Therapeutic Target in Human Cancers

**DOI:** 10.3390/ijms151018985

**Published:** 2014-10-20

**Authors:** Yanlan Yao, Jinming Li, Lunan Wang

**Affiliations:** 1 National Center for Clinical Laboratories, Graduate School of Peking Union Medical College, Beijing Hospital of the Ministry of Health, No. 1 Dahua Road, Beijing 100083, China; E-Mail: yaoylan@126.com; 2 National Center for Clinical Laboratories, Beijing Hospital of the Ministry of Health, No. 1 Dahua Road, Beijing 100083, China

**Keywords:** *HOTAIR*, lncrna, polycomb repressive complex 2, prognosis, therapeutic target

## Abstract

In the human genome, the fraction of protein-coding genes that are stably transcribed is only up to 2%, with the remaining numerous RNAs having no protein-coding function. These non-coding RNAs (ncRNAs) have received considerable attention in cancer research in recent years. Breakthroughs have been made in understanding microRNAs and small interfering RNAs, but larger RNAs such as long ncRNAs (lncRNAs) remain an enigma. One lncRNA, HOX antisense intergenic RNA (*HOTAIR*), has been shown to be dysregulated in many types of cancer, including breast cancer, colorectal cancer, and hepatoma. *HOTAIR* functions as a regulatory molecule in a wide variety of biological processes. However, its mechanism of action has not been clearly elucidated. It is widely believed that *HOTAIR* mediates chromosomal remodeling and coordinates with polycomb repressive complex 2 (PRC2) to regulate gene expression. Further study of *HOTAIR*-related pathways and the role of *HOTAIR* in tumorigenesis and tumor progression may identify new treatment targets. In this review, we will focus on the characteristics of *HOTAIR*, as well as data pertaining to its mechanism and its association with cancers.

## 1. Introduction

In the human genome, the fraction of protein-coding genes that are stably transcribed is only up to 2% [1]. Thus, a large number of transcripts do not encode proteins. These non-coding RNAs (ncRNAs) can be divided into housekeeping non-coding RNAs and regulatory non-coding RNAs. Housekeeping non-coding RNAs include ribosomal, transfer, small nuclear, and small nucleolar RNAs. They are usually constitutively expressed. The short regulatory ncRNAs (<200 nt) include microRNAs, small interfering RNAs (siRNAs), and piwi-associated RNAs. In addition, long non-coding RNAs (lncRNAs) [2,3], 200–100,000 nt in length, contribute to the regulation of gene expression at various levels, including chromatin modification, transcription, and post-transcriptional processing. Studies have shown that some lncRNAs are dysregulated in various cancers, contributing to tumorigenesis and tumor progression. They may have theranostic applications in human cancers. Among them, the lncRNA HOX antisense intergenic RNA (*HOTAIR*) was identified in 2007 [4], and numerous other lncRNAs have been discovered by next-generation sequencing and computational prediction. In this review, we summarize recent research on *HOTAIR* lncRNA, with a focus on its identification, characterization, mechanism of action, and association with related human cancers.

## 2. Mechanism of lncRNA Function and lncRNA-Related Cancers

### 2.1. Possible Origins of lncRNAs

**Figure 1:**
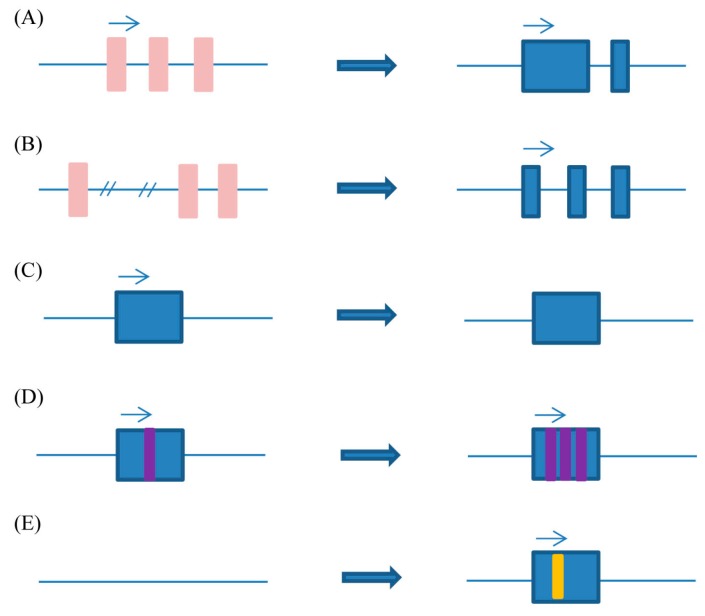
Possible origins of lncRNA. (**A**) acquire frame disruptions and transform into a functional noncoding RNA; (**B**) two untranscribed and previously well-separated sequence regions are juxtaposed through chromosome rearrangement; (**C**) generates either a functional noncoding retrogene or a nonfunctional noncoding retropseudo-gene, resulting from duplication of a noncoding gene by retrotransposition; (**D**) to occur neighboring repeats; (**E**) by inserting a transposable element.

Five kinds of possible origins of lncRNA are shown (Figure 1). First, a protein-coding gene (left, pink) acquires frame disruptions and is transformed into a functional noncoding RNA (right, blue) that incorporates some previous coding sequence. The *Xist* lncRNA originated by undergoing a metamorphosis from a previous protein-coding gene while incorporating transposable element sequence (Figure 1A); Secondly, following a chromosome’s rearrangement, two un-transcribed and previously well-separated sequence regions are juxtaposed and give rise to a multi-exon noncoding RNA. A dog noncoding RNA (supported by ESTs BM537447, C0597044, and DN744681) appears to have arisen following such a lineage-specific change (Figure 1B); Thirdly, duplication of a noncoding gene by retrotransposition generates either a functional noncoding retrogene or a nonfunctional noncoding retropseudo-gene (Figure 1C); Fourthly, Neighboring repeats within a noncoding RNA have their origins in two tandem duplication events (Figure 1D); Fifthly, insertion of a transposable element (yellow) gives rise to a functional noncoding RNA (Figure 1E) [4].

### 2.2. Mechanism of lncRNA Function

**Figure 2:**
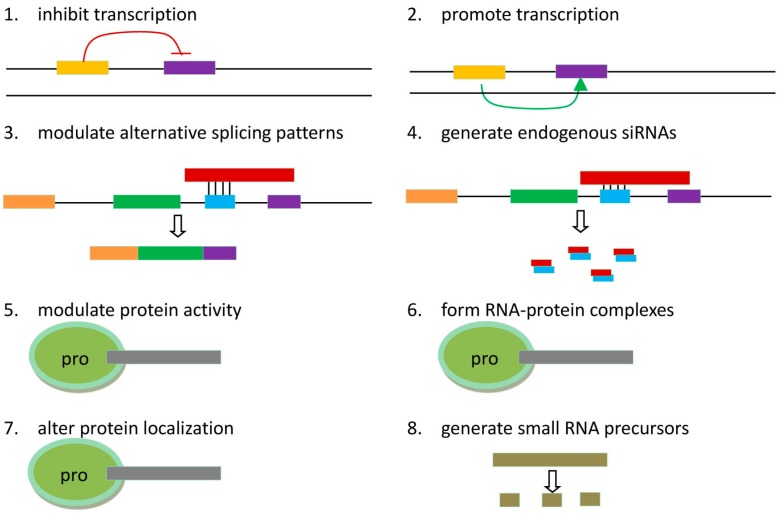
Possible mechanisms of lncRNA function. lncRNAs can (**1**) interfere with downstream gene transcription by inhibiting RNA polymerase II recruitment or (**2**) promote downstream gene expression by inducing chromatin remodeling and histone modifications; (**3**) An antisense lncRNA can modulate alternative splicing patterns by hybridizing to the complementary transcript; (**4**) Hybridization of lncRNA and mRNA allows Dicer to generate endogenous siRNAs; lncRNAs can bind to protein partners to (**5**) modulate protein activity; (**6**) serve as structural components; or (**7**) alter protein localization; (**8**) lncRNAs can generate small RNA precursors through certain processes.

Recent studies have shown that lncRNAs participate in various regulatory processes. Figure 2 outlines eight paradigms for how lncRNAs function.

### 2.3. Cancer-Related lncRNAs.

The evidence for lncRNA involvement in tumorigenesis includes findings that the lncRNA *MALAT1* (metastasis-associated lung adenocarcinoma transcript 1) promotes lung cancer metastasis [5]; participates in regulation of cell proliferation, migration, and invasion in colorectal cancer metastasis [6,7]; and is associated with an increased risk of liver tumor recurrence [8,9], and breast cancer metastasis [10].

*HOTAIR* is overexpressed in breast, colorectal, liver, and nasopharyngeal cancers [11,12,13,14,15]. Loss of *HOTAIR* moderates the invasiveness of breast cancer, particularly in cells in which polycomb repressive complex 2 (PRC2) is up-regulated. Similarly, *HOTAIR* also induces genome-wide PRC2 retargeting, participates in epigenetic regulation in colorectal cancer, and promotes metastases. In liver cancer, *HOTAIR* is a prognostic factor of hepatoma metastases and relapse after hepatic resection or liver transplantation. Moreover, up-regulated expression of *HOTAIR* is an indicator of poor prognosis in many other kinds of cancers [8,16,17,18,19,20,21].

Another lncRNA, highly up-regulated liver cancer (*HULC*), is reportedly overexpressed in hepatocellular carcinoma, other metastatic liver cancers [22,23] and liver cancer cell lines [24]. Up-regulated *HULC* may act as miRNA sponge and down-regulate *miR-372*, an important miRNA in liver cancer. This results in the phosphorylation of cAMP responsive element binding protein 1 (CREB1). Moreover, phosphorylated CREB1 participates in transcriptional inducement of *HULC* in the nucleus since it contains a CREB binding site in the core promoter. In other words, fine tuning of *HULC* expression is part of an auto-regulatory loop [24]. In addition to *HULC*, another lncRNA transcribed from gene *H19* promotes tumor growth in hepatocellular carcinoma [25].

*ANRIL* (antisense non-coding RNA in the *INK4* locus) was first identified through genetic analysis of familial melanoma patients [26]. *ANRIL* was also found to be associated with breast cancer [27,28,29], nasopharyngeal carcinoma, basal cell carcinoma, and glioma. Several reports have provided mechanistic insights into its molecular functions, which include binding to SUZ12 and recruitment of PRC2, but further studies are needed. Table 1 lists several cancer-associated lncRNAs and their functions.

**Table 1:** Cancer-related lncRNAs.

Cancer Type	lncRNA	Function	Reference
**Breast cancer**	*HOTAIR*	Chromatin remodeling	[11,30]
	*ANRIL*	Chromatin remodeling	[27,28,29]
	*MALAT1*	Gene relocation, scaffold, alternative splicing	[4,10]
**Colorectal cancer**	*HOTAIR*	Chromatin remodeling	[11,13]
	*HULC*	Liver metastasis	[23]
	*MALAT1*	Gene relocation, scaffold, alternative splicing	[7]
**Hepatocellular carcinoma**	*MALAT1*	Gene relocation, scaffold, alternative splicing	[4]
	*HULC*	MiRNA sponge	[23]
	*HOTAIR*	Chromatin remodeling	[31]
	*H19 lncRNA*	Promoting tumor growth	[25]

## 3. Characteristics and Mechanisms of *HOTAIR*

### 3.1. Identification and Characterization of HOTAIR

*HOTAIR* has attracted considerable attention from researchers worldwide. It was found to promote breast cancer metastasis and then shown to be pervasively overexpressed in most human cancers when tumor tissue was compared with adjacent noncancerous tissue. *HOTAIR* is a 2158 nt lncRNA discovered by Howard Chang’s group (lncRNA Database, http://www.lncrnadb.org/). An ultra-high-density *HOX* tiling array analysis showed that over 200 ncRNAs are transcribed from the four human *HOX* loci (*HOXA–HOXD*). *HOTAIR* is transcribed from the developmental *HOXC* locus located at chromosome 12q13.13, in a position intergenic and antisense to the flanking *HOXC11* and *HOXC12* genes [4,13,32,33]. *HOTAIR* induces the transcriptional silencing of the *HOXD* locus on chromosome 2 in trans [32,34]. It is the first lncRNA found to regulate gene expression in trans. Overexpression of *HOTAIR* has been found in breast cancer, colorectal cancer, and hepatoma.

Sequence analyses suggested that orthologues of *HOTAIR* are present only in mammals [33]. *HOTAIR* is located in the *HOXC* locus, but it has evolved faster than the neighboring *HOXC* genes. So, *HOTAIR* has poorly conserved sequences and considerably conserved structures. The cognate murine *HOTAIR* shows only 58% sequence identity to human *HOTAIR*. Deleting the *HOXC* cluster containing *HOTAIR* in mice produced no obvious effect and did not affect *HOXD10* expression, which suggests poor conservation of function [35]. However, a 239-bp domain within the 1804-bp exon 6 is well conserved and could be essential for *HOTAIR* function [33].

### 3.2. Mechanisms of HOTAIR Function

*HOTAIR* interacts with and recruits PRC2 and regulates the chromosome occupancy of EZH2 (a subunit of PRC2), which leads to histone H3 lysine 27 trimethylation of the *HOXD* locus. Moreover, *HOTAIR* can trigger the epithelial-mesenchymal transition (EMT) and interact with other small RNAs.

#### 3.2.1. *HOTAIR* Reprograms the Chromatin State through PRC2/LSD1

*HOTAIR* is the first lncRNA that has been found to act in trans. It is transcribed from *HOXC* but mediates the silencing of some tumor-suppressing genes of the *HOXD* locus. It is widely believed that PRC2, which comprises the EZH2 (Enhancer of Zeste 2, a histone methylase), SUZ12, and EED subunits, is essential for this silencing. *HOTAIR* recruits and interacts with PRC2 and regulates chromosome occupancy by EZH2 or SUZ12 [34] through its 5' domain, which leads to histone H3K27 methylation and chromosomal remodeling of the *HOXD* locus. In other words, *HOTAIR* induces genome-wide re-targeting of PRC2 and an altered gene expression pattern, promoting tumorigenesis and tumor progression [4,11,21,33], as described in Figure 2(**7**). Enforced *HOTAIR* expression in epithelial cancer cells increases invasive and metastatic abilities and reprograms the PRC2 occupancy pattern to resemble that of embryonic fibroblasts. Furthermore, *HOTAIR* function is not just limited to *HOXD*; it can recruit PRC2 to 854 genes that do not bind with PRC2 under normal conditions, resulting in gene overexpression or repression. Conversely, depletion of PRC2 proteins significantly reverses the influence of *HOTAIR*, providing another line of evidence that HOTAIR acts by coordinating with PRC2 [11]. As *HOTAIR* and PRC2 are mutually dependent, drugs targeting endogenous *HOTAIR* or inhibitors of *HOTAIR*–PRC2 interactions could serve as a new therapy strategy against tumors overexpressing polycomb proteins. Understanding the precise molecular mechanisms by which *HOTAIR* regulates PRC2 will be a critical first step in exploring this potential new avenue in cancer therapy.

The binding of *HOTAIR* to EZH2 also depends on EZH2 protein modification. EZH2 is phosphorylated by cyclin-dependent kinase 1 (CDK1) at threonine residues 345 and 487 in a cell-cycle–dependent manner. A phosphomimetic mutation at residue T345 was found to increase *HOTAIR* binding, implying that phosphorylation of EZH2 by CDK1 or CDK2 might enhance its function during the S and G2 phases in the cell cycle [34].

In addition to PRC2, the *HOXD* region is bound by CoREST/REST repressor complexes, which contain lysine-specific demethylase 1 (LSD1). This demethylase mediates enzymatic demethylation of dimethylated H3K4 (H3K4me2). *HOTAIR* has also been found to bind to the LSD1/CoREST/REST complex [32]. The 5' domain of *HOTAIR* binds to PRC2, whereas the 3' domain binds to the LSD1 complex. Thus, *HOTAIR* is a modular bifunctional lncRNA with distinct binding domains for a histone methylase and demethylase, and it serves as a scaffold for at least two distinct histone modification complexes. The modifications of these DNA-binding proteins by *HOTAIR* regulate global gene expression. The bifunctional role of *HOTAIR* might be required for coordinating histone modifications of H3K27 methylation and H3K4 demethylation for epigenetic gene silencing to promote metastatic processes [36].

#### 3.2.2. *HOTAIR* Triggers the EMT Process

The role of *HOTAIR* in the tumorigenesis of epithelial cancers involves EMT triggering and stemness acquisition, and suppression of *HOTAIR* may reverse the EMT process [37]. Padua Alves *et al*. [38] found that treatment with TGF-β1 increased *HOTAIR* expression and triggered the EMT program. Interestingly, ablation of *HOTAIR* expression by siRNA prevented the EMT program stimulated by TGF-β1, as well as the colony forming capacity of colon and breast cancer cells. Further, the colon cancer stem cell subpopulation (CD133^+^/CD44^+^) expressed much higher levels of *HOTAIR* than the non-stem cell subpopulation. However, the mechanism by which *HOTAIR* triggers the EMT has rarely been elucidated. In gastric cancer cells, the EMT may be triggered by *HOTAIR* through up-regulation of “*snail*” (a transcription factor involved in EMT). This is similar to the functional scenario of Figure 2(**2**) [38]. Certainly, further demonstration is needed for supporting this viewpoint.

#### 3.2.3. *HOTAIR* Interacts with Tumor Suppressor miRNAs

Recent studies suggest that lncRNAs interact with other classes of non-coding RNAs, including microRNAs (miRNAs), and modulate their regulatory activity [39]. lncRNA can serve as an miRNA sponge and interfere with the tumor suppressor function of protective miRNAs, resulting in tumorigenesis. Chiyomaru *et al*. [36] demonstrated that genistein inhibited prostate cancer (PCa) cell growth by down-regulating oncogenic *HOTAIR* and its interaction with the tumor suppressor *miR-34a. miR-34a* was up-regulated by genistein in PCa cells, whereas *HOTAIR* expression was down-regulated, suggesting that *miR-34a* plays an important role in the effects of genistein treatment. Furthermore, luciferase reporter assays confirmed the binding of *miR-34a* to its predicted binding site in *HOTAIR* [36], providing further support for their interaction.

#### 3.2.4. *HOTAIR* Competes with BRCA1

The *BRCA1* gene is directly associated with hereditary breast cancer, and its protein product, BRCA1, is normally expressed in the cells of the breast and other tissues. BRCA1 helps repair damaged DNA or destroy the cell if the DNA cannot be repaired. BRCA1 is involved in the repair of chromosomal damage, playing an important role in the error-free repair of DNA double-strand breaks. Moreover, in EZH2, the BRCA1-binding region overlaps with the *HOTAIR*-binding domain, and BRCA1 inhibits the binding of EZH2 to *HOTAIR*. Thus, decreased expression of BRCA1 causes genome-wide EZH2 retargeting and elevates H3K27me3 levels at PRC2 target loci, which play a role in *HOTAIR*-related breast cancers [40], as shown in Figure 2(**7**).

## 4. *HOTAIR*-Related Cancers

*HOTAIR* plays a role in tumorigenesis and tumor progression. In tumorigenesis, *HOTAIR* acts as an oncogene; in tumor progression, *HOTAIR* promotes invasion and metastases. However, the pathways and molecules that mediate *HOTAIR*ʼs effects must be understood before *HOTAIR* can be used in cancer treatment.

### 4.1. HOTAIR Is a Powerful Prognostic Factor in Breast Cancer (BC)

Dysregulation of *HOTAIR* in various cancers is associated with metastasis and tumor progression. *HOTAIR* was found to be highly up-regulated in primary, as well as metastatic, breast tumors, and its elevation correlated with both metastasis and poor survival rate [11,30].

*HOTAIR* expression is increased in primary breast tumors and metastases (as much as 2000-fold), and *HOTAIR* expression in primary tumors is a powerful predictor of eventual metastasis and death, independent of known clinicopathologic risk factors. Gupta *et al*. [11] measured *HOTAIR* levels in an independent panel of 132 primary breast tumors (stage I and II) with extensive clinical follow-up. Nearly one-third overexpressed *HOTAIR* by more than 125-fold when compared to normal breast epithelia. A high level of *HOTAIR* is a significant predictor of subsequent metastasis (*p* = 0.0004) and death (*p* = 0.005). Stable overexpression of *HOTAIR* by several hundredfold through retroviral transduction promoted colony growth in soft agar. Conversely, depletion of *HOTAIR* with siRNA in the MCF7 cell line, which expresses endogenous *HOTAIR*, decreased matrix invasiveness, thus demonstrating the function of *HOTAIR in vitro*. In addition, Gupta *et al*. labeled control and *HOTAIR*-expressing MDA-MB-231 cells with firefly luciferase and grafted the cells into mammary fat pads. Two weeks later, the number of luciferase foci in the lung fields was higher in mice with *HOTAIR*-expressing primary tumors than in control mice, which suggests that *HOTAIR* promotes lung metastasis *in vivo*.

#### 4.1.1. *HOTAIR* and the Methylation Status of Down-Stream CpG Islands

The prognostic value of *HOTAIR* in breast cancer was widely accepted until an article published at the end of 2012 challenged the prevailing view. Lu *et al*. [41] analyzed DNA methylation in 348 primary breast cancer tissues with methylation-specific PCR and found a positive correlation between DNA methylation and *HOTAIR* expression. Methylation was associated with unfavorable disease characteristics. However, no significant associations were found between *HOTAIR* expression and clinical or pathologic features. Patients with high *HOTAIR* expression had lower risks of relapse and mortality than those with low *HOTAIR* expression. These findings suggest that intergenic DNA methylation may be important in regulating *HOTAIR* expression and that *HOTAIR* expression may not be an independent prognostic marker in breast cancer. However, the findings require further validation in independent studies.

#### 4.1.2. *HOTAIR* Function in Breast Cancer Is Associated with Estrogen Receptor Status

Recent studies showed that *HOTAIR* is an independent prognostic factor of metastases in estrogen receptor (ER)-positive primary breast cancer. A retrospective study of 164 primary breast cancer patients by Sørensen *et al*. [15] found that a high level of *HOTAIR* was significantly associated with worse prognosis (*p* = 0.012). The association was even stronger when only ER-positive tumor samples were considered (*p* = 0.0086). Bhan *et al*. [42] demonstrated that *HOTAIR* requires transcriptional induction by E2. The *HOTAIR* promoter contains multiple functional estrogen response elements. In the presence of E2, ERs and various ER coregulators bind to the promoter. Meanwhile, the level of H3K4 trimethylation, histone acetylation, and RNA polymerase II recruitment is enriched at the *HOTAIR* promoter. In contrast, knockdown of ERs down-regulates E2-induced *HOTAIR* expression. Given these findings, therapeutic methods that target *HOTAIR* or regulate ER status may help suppress the progression of breast cancer.

### 4.2. HOTAIR Is a Potential Prognostic Factor in Gastroenteric Tumors

As in breast cancer, *HOTAIR* also induces genome-wide PRC2 retargeting, participates in epigenetic regulation and promotes metastases in colorectal cancer (CRC) [11,13,43]. Analysis of approximately 100 cancer tissues showed that *HOTAIR* expression was significantly higher in cancer tissues than in normal tissue samples (*p* = 0.002). After dividing the cancer tissues into a high *HOTAIR* expression group (*n* = 20) and a low expression group (*n* = 80) using a *HOTAIR/GAPDH* ratio of 0.273, further analysis revealed that the high expression group exhibited less differentiated histology (*p* = 0.024), greater tumor depth (*p* = 0.039), greater liver metastases (*p* = 0.006), and worse prognosis (*p* = 0.0046). Transduction of lentiviral vectors encoding *HOTAIR* into the HCT116 cell line promoted cell invasion in Matrigel (*p* < 0.05). Conversely, siRNA-induced knockdown of *HOTAIR* decreased cell invasion (*p* < 0.05), further suggesting that *HOTAIR* is related to liver metastases and is a potential prognostic factor in colorectal cancer [13]. On the other hand, *HOTAIR* suppression sensitized cancer cells to tumor necrosis factor α (TNF-α), induced apoptosis, and rendered the cells more sensitive to the chemotherapeutic agents cisplatin and doxorubicin [13], indicating that *HOTAIR* could be a target for therapy.

Svoboda M, *et al*. [43] also assessed the prognostic value of *HOTAIR* expression. On one hand, they underlined the prognostic potential of *HOTAIR* expression level in tumor tissues of CRC patients, both in univariate analysis (*p* = 0.046) and multivariate analysis (*p* = 0.048). What is more, they demonstrated that *HOTAIR* relative expression in tumor and paired blood are positively correlated (*R* = 0.43, *p* = 0.03). In univariate analysis, *HOTAIR* levels in blood were associated with higher mortality of patients (Cox’s proportional hazard, hazard ratio = 5.9, 95% confidence interval: 1.3–26.1, *p* = 0.019). This means that the *HOTAIR* expression in blood can also be an independent prognostic marker in CRC. As a kind of surrogate sample of tumor tissue, the non-invasively obtained HOTAIR data may have vast clinical potential in prognosis. Moreover, HOTAIR overexpression-either in tumor tissue or in blood-may identify patients that would require more intensive care of personally tailored treatment.

EZH2 and SUZ12, the components of PRC2, are overexpressed in several cancers. In particular, SUZ12 is reportedly overexpressed in colorectal cancer. Gene pathway analysis indicated that *HOTAIR*-regulated gene sets included CDH1 (E-cadherin) target genes, whose expression is lost in metastatic cancer cells of the mesenchymal phenotype. Thus, *HOTAIR* might cooperate with PRC2 to maintain mesenchymal and undifferentiated cancer cells. For these reasons, *HOTAIR* or SUZ12 might be a treatment target in colorectal cancer.

Studies of *HOTAIR* in gastric cancer (GC) have just begun, but its relevance has already been demonstrated. Using soft agar assays, Endo *et al*. [17] showed that the anchorage-independent growth of gastric cancer cells depended on *HOTAIR* expression. They grafted gastric cancer cells with increased or suppressed *HOTAIR* expression into the tail vein or peritoneal cavity of immunodeficient mice. The *HOTAIR*-expressing group showed greater venous invasion, more frequent lymph node metastases, and lower overall survival rate than the *HOTAIR*-suppressed group. *HOTAIR*-expressing cells were also more likely to induce liver metastases. These findings suggest that *HOTAIR* has prognostic value in gastric cancer *in vitro* and *in vivo*. However, the molecular mechanism of *HOTAIR* in gastric cancer is poorly understood. Recently published data suggested it may function as a miRNA sponge and induces down-regulation of *miR-331-3p*, thereby modulating the derepression of *HER2* expression and promoting migration and invasion of gastric cancer cells [44,45]. Meanwhile, Xu *et al*. [37] found that inhibition of *HOTAIR* could reduce invasiveness and reverse EMT process in these cells. These results also indicate that *HOTAIR* may be a therapeutic target in the treatment of gastric cancer.

### 4.3. HOTAIR Is a Potential Prognostic Factor of Hepatoma Metastases and Relapse after Hepatic Resection or Liver Transplantation

In primary hepatocellular carcinoma (HCC), *HOTAIR* overexpression is not observed in every cancer patient. However, a study of HCC patients showed that patients with *HOTAIR* overexpression exhibit larger tumor size, worse prognosis, and an increased risk of metastasis when compared to patients with normal *HOTAIR* levels [12]. In a study of 63 patients after hepatectomy, Geng *et al*. [46] found that *HOTAIR* was associated with hepatocellular growth and concluded that *HOTAIR* might be a biomarker for predicting lymph node metastasis. In some cancer patients, the *HOTAIR* level of the cancer biopsy was higher than adjacent normal tissue; and this cohort showed higher risk of relapse. Knockdown of *HOTAIR* down-regulated proteins related to cell motility and metastasis, such as matrix metalloproteinase (MMP)-9 and vascular endothelial growth factor (VEGF), and decreased proliferation of Bel7402, a hepatoma cell line. On the other hand, *HOTAIR* levels were increased in tumor samples from patients with lymph node metastasis.

In some patients who underwent liver transplantation, Yang *et al*. [31] also observed higher *HOTAIR* expression in cancer tissues than in adjacent normal tissues and found that *HOTAIR* was an independent prognostic factor for relapse (*p* = 0.001). The survival period after relapse was shorter in these patients. Similarly, in a hepatoma cell line, interfering *HOTAIR* function with specific siRNA affected viability and invasiveness and increased the sensitivity to TNF-α-induced apoptosis and cisplatinum/doxorubicin treatment. Thus, after liver transplantation, *HOTAIR* is a promising prognostic factor and a potential target for treatment.

Recently, the research team of Yang reported their study on the role and molecular mechanism of *HOTAIR* in HCC progression. They found that *HOTAIR* suppression significantly increased the expression of RNA binding motif protein 38 (RBM38), and the expression levels of RBM38 were significantly lower than adjacent normal tissues. Moreover, RBM38 suppression by siRNA strategy also reversed the cell migration and invasion compared with *HOTAIR*-knockdown cells [47]. These results suggest that RBM38 is one of the direct target of *HOTAIR* in HCC progression, and underlines the potential of *HOTAIR* as a therapy target.

VEGF and MMP-9 play important roles in hepatocellular carcinoma progression. Because these proteins are regulated by *HOTAIR*, treatments that target *HOTAIR* might suppress growth. On the other hand, drugs that target VEGF or MMP-9 directly might also be helpful for hepatoma treatment.

In summary, the lncRNA *HOTAIR* is important in breast, gastroenteric, and liver cancers. The signaling molecules most likely to interact with *HOTAIR* in these cancers are shown in Table 2, and some of the related pathways that are most likely to be applied to therapeutic strategy are enumerated in Table 3, Table 4, Table 5 and Table 6.

**Table 2:** *HOTAIR*-related signaling molecules.

Cancer Type	lncRNA	Related Molecules	Reference
BC	*HOTAIR*	PRC2/LSD1/E2/BRCA1	[11,30]
CRC	*HOTAIR*	EZH2/SUZ12/CDH1	[11,13]
HCC	*HOTAIR*	VEGF/MMP-9	[31]

**Table 3:** *HOTAIR*-related signaling pathways/processes in BC.

Pathways	Signaling Molecules	Biological Processes
ErbB signaling pathway	*HER2*	cell migration/evasion
NF-kappa B signaling pathway	*HER2*	DNA degradation/cell survival
PI3K-Akt signaling pathway	BRCA1/EZH2	DNA repair/cell proliferation; angiogenesis
TGF-β signaling pathway	*twist/snail/miR-10b*	EMT process; Cell growth/survival; Cell migration/invasion
estrogen signaling pathway	E2	Cell cycle/cell adhension; apoptosis
ubiquitin mediated proteolysis	BRCA1/E2	Degradation of target protein

**Table 4:** *HOTAIR*-related signaling pathways/processes in CRC.

Pathways	Signaling Molecules	Biological Processes
TGF-β signaling pathway	*snail*	EMT process
P53 signaling pathway	EZH2/CDK1	Cell cycle arrest
Cell cycle	CDH1/EZH2/CDK1	Cell cycle arrest
Ubiquitin mediated proteolysis	CDH1	Degradation of target protein

**Table 5:** *HOTAIR*-related signaling pathways/processes in GC.

Pathways	Signaling Molecules	Biological Processes
TGF-β signaling pathway	*snail*	EMT process
RNA interfere	*miR-331-3p*	miRNA sponge/*HER2*-mRNA protection; cell signaling networks

**Table 6:** *HOTAIR*-related signaling pathways/processes in HCC.

Pathways	Signaling Molecules	Biological Processes
VEGF signaling pathway	VEGF	cell proliferation/migration; sustained angiogenesis
TNF signaling pathway	MMP-3/MMP-9	remodeling of extracellular matrix
PPAR signaling pathway	MMP-1	adipocyte differentiation
Wnt signaling pathway	MMP-7	cell cycle
mRNA surveillance pathway	RBM38/p53	gene overexpression
TGF-βsignaling pathway	*miR-10b*	EMT process; cell growth and survival; cell migration and invasion

### 4.4. Other HOTAIR-Related Cancers

A relationship between *HOTAIR* and other types of tumors, including lung cancer, prostate cancer, pancreatic carcinoma, and sarcoma, has been reported. In non-small cell lung cancer, the effect of *HOTAIR* on cell invasion and metastases is induced by *HOXA5* [48]. Loss of *HOTAIR* in non-small cell lung cancer cells led to a significant decrease in the levels of MMP-2 and MMP-9, which are important for many biology processes, including cell proliferation, differentiation, remodeling of the extracellular matrix, vascularization, and cell migration. In lung adenocarcinoma, *HOTAIR* overexpression and p21 are responsible for cisplatinum resistance [49]. Depletion of *HOTAIR* by siRNA led to decreased invasion and increased apoptosis in HepG2 cells, and tumor growth was significantly inhibited in mice injected with *HOTAIR*-deficient cells [50]. Overexpression of *HOTAIR* is also associated with high-grade tumor and metastasis in gastrointestinal stromal tumors; knockdown of *HOTAIR* altered the expression of some reported target genes and suppressed cell invasiveness [51].

## 5. Conclusions

*HOTAIR* is dysregulated in some cancers. It plays a role in tumorigenesis and tumor progression, and it has prognostic value in BC, CRC, and HCC. Treatments that target *HOTAIR* are a new area of tumor research. However, large-scale and multicenter randomized tests are needed to confirm the value of *HOTAIR* as a prognostic factor. Meanwhile, to develop therapeutic applications with *HOTAIR* as a target, the pathways and molecules downstream of *HOTAIR* must be elucidated. Research into *HOTAIR* will hopefully lead to theranostic applications in human cancer.

Anti-tumor strategies that focus on RNA as a target molecule are currently under development. For cancer therapy, the cancer- and tissue-specific expression of lncRNAs is an advantage not offered by other therapeutic options. The use of HOTAIR in cancer therapy will require detailed knowledge about tumor-specific ncRNA function and cancer cell properties.

Theoretically, *HOTAIR* activity could be blocked in multiple ways. First, one could block molecular interactions using small molecule inhibitors that mask the binding site in protein interaction partners or antagonistic oligonucleotides that bind to the ncRNA and interfere with protein binding, thus silencing *HOTAIR*. Second, one could disrupt the secondary structure of *HOTAIR* using small molecule inhibitors or mimics to compete at the binding site. Third, *HOTAIR* could be silenced with specific siRNAs or redundant miRNAs. In addition to blocking *HOTAIR*, drugs could be developed that directly target molecules in the *HOTAIR* pathway listed in Table 3, Table 4, Table 5 and Table 6, such as ERs in BC, SUZ12 in CRC, and VEGF/MMP-9 in HCC. Meanwhile, further research into the *HOTAIR* pathway and related molecules is needed. *HOTAIR* shows great prognostic and therapeutic promise for various kinds of cancer.
